# Registration of phase 3 crossover trials on ClinicalTrials.gov

**DOI:** 10.1186/s13063-020-04545-2

**Published:** 2020-07-06

**Authors:** Lijuan Zeng, Riaz Qureshi, Shilpa Viswanathan, Lea Drye, Tianjing Li

**Affiliations:** 1 Statistics Collaborative, Inc, Washington, D. C. USA; 2 Center for Clinical Trials and Evidence Synthesis, Department of Epidemiology, Johns Hopkins Bloomberg School of Public Heath, Baltimore, MD USA; 3 IQVIA, Parsippany, NJ USA; 4 Blue Cross Blue Shield Association, Chicago, IL USA; 5 Department of Ophthalmology, School of Medicine, University of Colorado Denver, Aurora, CO USA

**Keywords:** Crossover trials, Trial registration, ClinicalTrials.gov, Longitudinal trial, Data sharing, Trial reporting

## Abstract

**Background:**

In a randomized crossover trial, each participant is randomized to a sequence of treatments and treatment effect is estimated based on within-individual difference because each participant serves as his/her own control. This feature makes the design and reporting of randomized crossover trials different from that of parallel trials. Our objective was to characterize phase 3 crossover trials with results reported on ClinicalTrials.gov and identify issues and best practices for reporting.

**Methods:**

We searched ClinicalTrials.gov for phase 3 randomized crossover trials that provided results, registered at least one primary outcome, and included at least one link to a results publication in the record by August 6, 2019. Two reviewers independently assessed the eligibility and extracted information from each record into an electronic form developed and maintained in the Systematic Review Data Repository.

**Results:**

Of the 124 crossover trials analyzed, two thirds were a simple “Intervention A then B” or “Intervention B then A” (AB|BA) design. Most trials (78%, 97/124) provided enough information to understand the participant flow throughout the trial. Baseline characteristics were most often reported for all participants as a single group (52%, 65/124). Primary outcomes and adverse events were most commonly reported “per intervention” (85%, 105/124, and 80%, 99/124, respectively).

**Conclusions:**

The registration and reporting of randomized crossover trials must account for the paired nature of the design. Our observations and recommendations informed the development of guidelines for good reporting practices in the registration and reporting of randomized crossover trials.

## Background

ClinicalTrials.gov is a web-based, publicly accessible clinical trial registry maintained by the National Library of Medicine of the National Institutes of Health. Since its advent in 1997, ClinicalTrials.gov has emerged to become the primary registry for federally and privately funded clinical trials conducted in the USA [1].

Trials registered on ClinicalTrials.gov utilize different designs, with parallel- or single-group assignment being the most frequently utilized designs [2]. In a parallel, randomized controlled trial, each participant is randomized to receive one treatment (for example, treatment A or treatment B) and treatment effect is estimated via comparison of outcome measures between the two independent groups of participants. In contrast, in a randomized crossover trial, participants receive multiple treatments and are randomly assigned to a specific sequence (i.e., the order in which treatments are received, for example, treatment A then treatment B or vice versa), and estimation of treatment effect needs to account for the correlation of repeated measurements in the same participant, as each participant serves as his/her own control [3]. A major advantage of using a crossover design can be the removal of the between-subject variation in estimating treatment effects. In most cases, the sample size needed to detect a fixed effect size is smaller with a crossover design compared to a parallel design [4].

Because the crossover design requires repeated measurements on the same participant in multiple periods with different interventions, special attention is needed in the design, analysis, and reporting of a crossover trial. A crossover design can potentially have carryover effects: persistent or residual effects from treatment in one period that may confound the estimated treatment effect in subsequent treatment periods. For example, if the effect of treatment A from period 1 persists to period 2, in which a participant is receiving treatment B, the treatment effect observed in the second period will be a combined effect from both treatments yet, attributed only to treatment B. Another potential issue in crossover trials is the period effect, which occurs when secular changes are present: For example, a period effect may arise if a condition under study is not stable, such that the effects of treatment are not consistent over time and the true effects of each intervention are confounded by the period in which they are received. Additionally, in terms of missing data, even a small amount of missing data in a crossover trial can result in a compromised study [3]. Finally, the randomized crossover design is inappropriate for conditions in which the treatment in an earlier period permanently alters the course of the condition (e.g., cure the disease), such that at the entry to the next period, the participant characteristics systematically differ from their initial states at the start of the trial [4–6].

Because of these important and distinct features and challenges, the design, analysis, and reporting of crossover trials are different from that of parallel trials [5]. Recent studies evaluating the characteristics of a large number of crossover trials have shown that the design and reporting of crossover trials in journals were largely inadequate and their respective analyses inappropriate, hence limiting their value to inform clinical practice [6, 7]. None of these studies have described crossover trials with results reported on ClinicalTrials.gov, an increasingly valuable database in which investigators provide key design features and the results of their clinical trials [8]. Our objectives are to characterize phase 3 crossover trials with results reported on ClinicalTrials.gov and identify issues and best practices for reporting.

## Materials and methods

### Trial selection

We included randomized, phase 3 crossover trials registered on ClinicalTrials.gov that (1) had results reported, (2) registered at least one primary outcome, and (3) included at least one link to a results publication by August 6, 2019. In the case when the ClinicalTrials.gov registry provided insufficient information to determine if the trial was a randomized crossover trial, we referred to citations provided by trial sponsors or investigators, indexed in the “Publication” section on ClinicalTrials.gov. We chose to focus on phase 3 trials as a starting point because phase 3 trials are more likely to have results registered on ClinicalTrials.gov and more likely to be published in the scientific literature than earlier phase trials (i.e., phases 1 or 2) [9]. We considered trials to be “phase 3” if they were classified as such by the investigators in clinicaltrials.gov. We included only individual randomized crossover trials (i.e., cluster randomized crossover trials would be excluded).

To identify trials meeting our eligibility criteria, we retrieved records through the following search elements: “Other terms” = “Crossover assignment” OR “Crossover” OR “Cross-over”; “Study type” = Interventional studies (Clinical Trials); “Study Results” = Studies With Results; “Phase” = Phase 3. Our first check for eligibility was that the “Intervention model” was “Crossover design,” followed by the verification of phase and randomization, and presence of a publication. Intervention model is a term used on ClinicalTrials.gov to describe “the general design of the strategy for assigning interventions to participants in a clinical study [https://clinicaltrials.gov/ct2/about-studies/glossary#I].” Types of intervention models include single-group design, parallel design, crossover design, and factorial design. The purpose of selecting records with linked citations/publications was to provide an additional source of trial characteristics to assess eligibility for our analysis if insufficient or unclear information was available on ClinicalTrials.gov. For this study, two individuals independently assessed each record for eligibility, consulting the linked publications as needed for supplemental information.

### Data abstraction

For eligible records, we first downloaded all publicly available data fields from ClinicalTrials.gov. In addition, two individuals independently reviewed each eligible record on the ClinicalTrials.gov website and abstracted information from the protocol and results sections of each registration record into an electronic data collection form developed and maintained in the Systematic Review Data Repository (SRDR) [10]. We extracted data solely from ClinicalTrials.gov—the requirement of an included trial to have a linked publication was to determine if the trial is a randomized crossover trial. Investigators can update a record registered on ClinicalTrials.gov at any time. We used information current to August 6, 2019, for this study. We resolved data abstraction discrepancies between the two reviewers by consulting a third investigator on the team.

### Descriptions of data abstracted from the protocol section

The protocol section of a study record on the ClinicalTrials.gov contains information about the study design, recruitment status, study sponsor, and contact details. For this study, we focused on the data elements “Arms” and “Assigned Interventions,” which are most relevant for accurately describing the crossover design. Investigators use “Arms” and “Assigned Interventions”—sections of the registration in ClinicalTrials.gov—to describe the intervention strategy used in the trial. “Arms” provides the name for identifying the study groups and a brief description for each study group to distinguish it from other groups in the trial. “Assigned Interventions” specifies the interventions used for that arm.

**Fig. 1:**
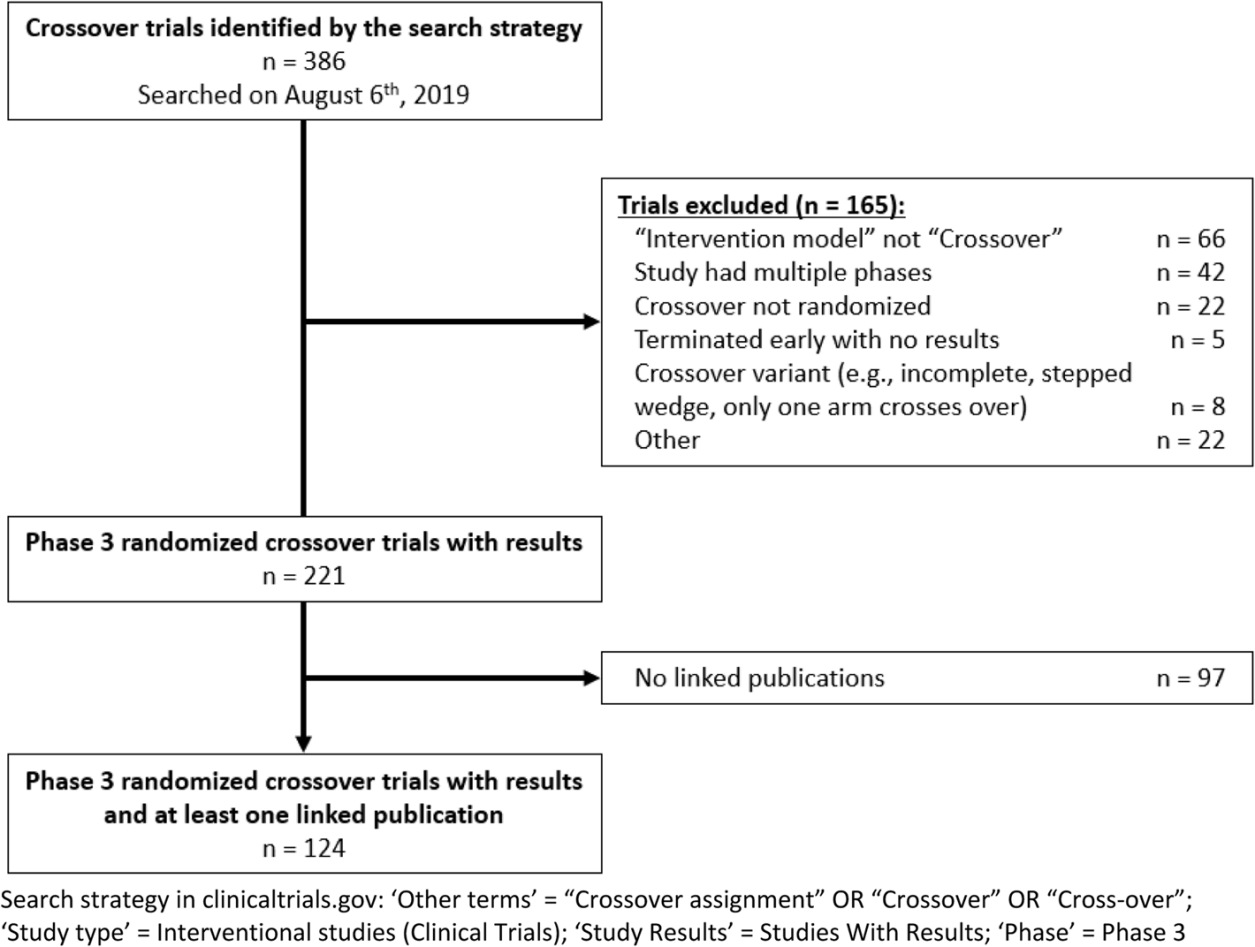
Records selection flow chart. Search strategy in clinicaltrials.gov: “Other terms” = “Crossover assignment” OR “Crossover” OR “Cross-over”; “Study type” = Interventional studies (Clinical Trials); “Study Results” = Studies With Results; “Phase” = Phase 3

We assessed whether information provided in the “Arms” and “Assigned Interventions” described the crossover design “by sequence” or “by intervention” (illustrations for each categorization are shown in S Figure 1) [11, 12]. Because in a randomized crossover trial, participants are randomized to a treatment sequence, the “by sequence” description of the study “Arms” properly illustrates the design. We also categorized the type of crossover design by reviewing the trial description, arms description, and treatment assignments. For example, a simple randomized crossover design comparing two treatments, with two periods and therefore two sequences, was categorized as “AB|BA” design (i.e., “A first, then B” or “B first, then A”).

### Descriptions of data abstracted from the results section

**Fig. 2:**
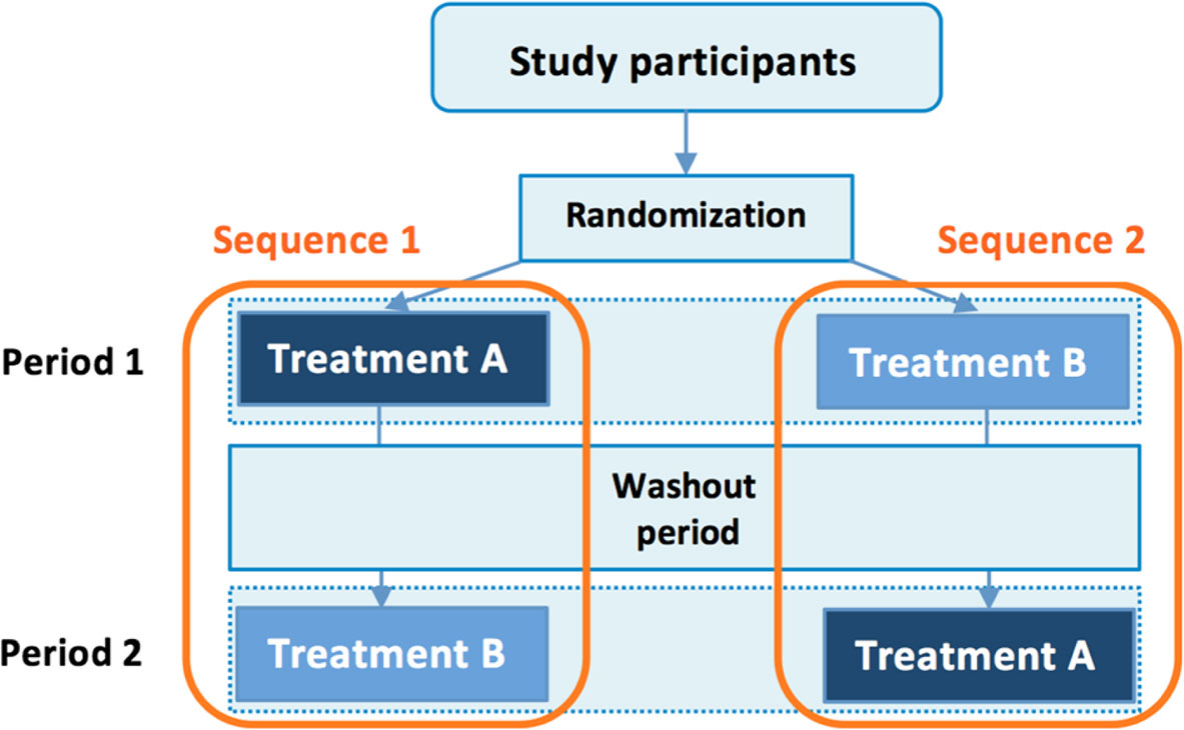
Illustration of the design and analysis of a randomized crossover trial

The results section of a study record on ClinicalTrials.gov contains scientific and administrative information about the results of the trial categorized by modules. For this study, we reviewed all four scientific modules: Participant Flow, Baseline Characteristics, Outcome Measures and Statistical Analyses, and Adverse Events. Each results module includes information reported in table format, with columns representing the assignment or comparison groups (“Reporting Groups”) and the rows representing the reported data. For each module, we recorded whether the reporting groups were by sequence, by intervention, by period, or by total only. In general, representing the reporting groups for each module by sequence allows for a clear representation of the crossover design and study population. In the Participant Flow module, we also recorded if more than one table was used to describe each period of the trial and if each table included enough information to determine the number of participants who started and dropped out within each period (see S Figure 2) [13, 14]. Another way to communicate participant flow through periods is by using “milestone” rows within one table.

For the Outcome Measures module, we abstracted data from the first primary outcome (i.e., the sole or first-listed primary outcome if there were multiple specified “primary outcomes”). In addition to categorizing the reporting groups, we classified each outcome by a specific metric, method of aggregation, specified time frame, and outcome type (e.g., continuous outcome, categorical outcome, and time-to-event outcome), following the framework proposed by Zarin et al. [15]. We also examined whether the quantitative results were provided for each period separately, which allows for understanding the likelihood of carryover effect and period effect. For the Adverse Events module, we focused on the reporting groups in the Serious Adverse Events table.

### Data analyses

We exported extracted data from SRDR for analysis. We tabulated the categorical distribution for each trial characteristic using STATA 13**®**. We did not conduct any hypothesis testing or estimation.

## Results

We identified 386 Phase 3 interventional trials with results and the “crossover” keyword via our search strategy on August 6, 2019. Of these, we excluded 66 which did not have “Crossover Assignment” for the intervention model, 42 for having multiple phases (e.g., phase 2|3 studies), and 22 for having a crossover that was not randomized. We further excluded 5 trials which were terminated early and did not present any results, 8 for being some variant of a crossover design (e.g., incomplete block in which participants do not receive all treatments, a stepped wedge design whereby the timing of arms crossing over is randomized, and only having a single arm cross over), and 22 for other reasons (e.g., unclear if there was any crossover between interventions). We identified 221 phase 3 randomized crossover trials with results, and of these, 97 did not have any linked publications. Thus, we included 124 phase 3 randomized crossover trials with results and at least one publication in our subsequent data abstraction (Fig. 1).

### Trials characteristics

The 124 randomized crossover trials in our analysis tested interventions for a variety of conditions, ranging from dental care, asthma and chronic obstructive pulmonary disorder, pain, migraine, prostate cancer, to chronic kidney disease and diabetes. The majority of trials (89%, 110/124) assessed drugs (S Table 1), and 80% (99/124) of included trials were funded in part or in whole by industry.

### Protocol section

#### Crossover sequence

Of the included trials, 68% (85/124) used a simple “AB|BA” crossover design, and 15% used a balanced and complete block of either six sequences “ABC|ACB|BCA|BAC|CAB|CBA” (16/124) or three sequences (2/124) such as “ABC|BAC|CAB” [16] (Table 1). Fifteen percent (17/124) of trials used designs with extra treatment periods—such as “ABA|BAB” [17] and “AAB|ABA|BAA” [18, 19]—or other sequences including three studies which used more than 6 sequences for more than two interventions: 8 sequences in a trial of 574 participants [20], 12 sequences in a trial of 96 participants [21], and 18 sequences in a trial of 54 participants [22]. Four trials (3%) did not register any sequences.

**Table 1:** Registration of key features for randomized crossover design (*N* = 124)

Characteristics^**a**^	***n***	(%)
**Protocol section**		
**Crossover sequence**		
AB|BA	85	(68)
ABC|ACB|BCA|BAC|CAB|CBA	16	(13)
AAB|ABA|BAA	2	(2)
ABC|BAC|CAB	2	(2)
ABA|BAB	1	(< 1)
ABCD|BDAC|DCBA|CADB	2	(2)
Others	12	(10)
No sequences registered	4	(3)
**“Arms” and “Assigned Interventions”**		
By sequence	73	(59)
By intervention	49	(39)
Others	2	(2)
**Results section**		
**“Participant Flow”**		
Provided sufficient information to understand the participant flow^b^	97	(78)
By sequence and used separate tables/periods	89	
By sequence and used “milestones” rows within one table	5	
By sequence and used one table, without missing data	2	
By intervention and used separate tables	1	
Provided insufficient information to understand the participant flow	27	(22)
By sequence and by total	18	
By total only	4	
Others	5	
**“Baseline Characteristics”**		
By sequence	55	(44)
By total only	65	(52)
Others	4	(3)
**“Outcome Measures”—first primary outcome**^b^		
By sequence	6	(5)
By sequence and by period	2	(2)
By intervention	105	(84)
By total only	3	(2)
Others	8	(6)
**“Serious Adverse Events”**		
By sequence	8	(6)
By total only	4	(3)
By intervention only	99	(80)
By intervention and period	3	(2)
Others	5	(4)
No adverse events reported	5	(4)

^a^Assessment was based on data presented on ClinicalTrials.gov on August 6, 2019

^b^Records that provided sufficient information to understand the participant flow may (1) use separate tables for each treatment period (e.g., NCT00432744); (2) use “milestone” rows for each period within one table (e.g., NCT00432835); (3) use one table when there is no missing data (e.g., NCT01808755); or (4) use separate tables for each treatment period by intervention (e.g., NCT01323660)

#### “Arms” and “Assigned Interventions”

Whereas 59% (73/124) of crossover trials described the study design by sequence in the “Arms” and “Assigned Interventions” fields, 39% (49/124) described the design by intervention, similar to a parallel design trial (Table 1). Of the remaining two trials classified as other (2%, 2/124), one trial reported by intervention in the title, but by sequence in the description paragraph [23]; the other trial reported by population based on a certain genotype [24].

## Results section

### “Participant Flow”

Seventy-eight percent (97/124) of trials provided enough information to understand the number of participants assigned to each sequence and starting and completing each period of the trial (Table 1). Among these 97 trials, 92% (89/97) had reporting groups by sequence with separate tables for each study period. Five trials had milestones (rows) within one table to describe the study periods [20, 25–28], two trials included only one table without separating the participant flow by period because there was no missing data [29, 30], and one trial presented their flow by intervention using separate tables [31]. The remaining trials (22%, 27/124) did not provide enough information to understand the numbers of participants starting and completing each intervention/period. Of these 27 trials, 18 presented the flow by sequence, but in a single table for the study overall, four presented the flow as a single table for the total enrollment and study duration, and five presented the flow by other methods.

### “Baseline Characteristics”

Forty-four percent (55/124) of trials described baseline characteristics by sequence (Table 1), which allows the assessment of the distribution of important prognostic factors between randomized sequences. Alternatively, 52% (65/124) of trials presented a “Total” column *only* (combining all randomized participants into a single group) for the Baseline Characteristics. For those trials that used a Total column, there were variations in terminology used to describe the group, such as “Overall Study Population,” “Entire Study Population,” “All participants,” and “Safety Population.” The remaining trials reported baseline characteristics by intervention for the whole study or for the first period, or used a study population based on genotype as reporting groups (3%, 4/124) [24, 32–34].

### “Outcome Measures”

The number of primary outcomes registered ranged from one to nine with 80% (99/124) of trials reporting one primary outcome, and three trials reporting more than five primary outcomes [13, 24, 35] (Table 2). Primary outcomes were summarized using a continuous measure in 81% (101/124) of trials, categorical measure in 14% (17/124), 3% (4/124) using a time-to-event measure, and the remaining 1% (2/124) using other measures (diagnostic yield for neoplasia [36] and total number of adverse events [37]) (Table 2). Seventy-eight percent (97/124) of trials presented results for participants from all randomized crossover periods as opposed to a subset of periods (Table 2). Three percent (4/124) of the trials presented period-specific results, which can be used to check whether carry over effect and/or period effect is likely. Sixty-three percent (78/124) of the trials provided a description of the statistical analysis on ClinicalTrials.gov.

**Table 2:** Summary of outcome measures and characteristics of the first primary outcome (*N* = 124)

Characteristics	***n***	(%)
**Number of primary outcome(s)**		
1	99	(80)
2	16	(13)
3	6	(5)
> 3	3	(3)
**Types of data reported for the primary outcome**^**a**^		
Continuous outcome	101	(81)
Categorical outcome	17	(14)
Time-to-event outcome	4	(3)
Others	2	(1)
**Specific metric for the primary outcome**		
Value at a time-point	74	(60)
Time-to-event	4	(3)
Change from the period-baseline	31	(25)
Within individual difference between values at the end of each period	5	(5)
Within individual difference between changes from baseline	1	(1)
Others	8	(6)
**Method of aggregation for the primary outcome**		
Number—count of participants	21	(17)
Mean	50	(40)
Median	4	(3)
Least squares mean	43	(35)
Number—proportion/percent	6	(5)
**Time frame for the primary outcome includes all randomized periods**		
Yes	97	(78)
No	27	(22)
**Measured values presented by period**		
Yes	4	(3)
No	120	(97)
**Provides statistical analysis for primary outcome**		
Yes	78	(63)
No	46	(37)
**Statistical methods for analyzing primary outcome (*n*** **= 78)**		
ANCOVA	9	(11)
ANOVA	14	(18)
Cochran-Mantel-Haenszel	2	(3)
Generalized estimating equations	2	(3)
Generalized linear mixed model	3	(4)
Linear mixed effects model	6	(8)
Mixed model analysis	20	(26)
Mixed effects ANOVA crossover model	3	(4)
Non-inferiority/equivalence test^b^	2	(3)
Prescott’s test	1	(1)
*t* test	3	(4)
Two-sided signed-rank test	1	(1)
Wilcoxon (Mann-Whitney)	5^b^	(6)
Wilcoxon signed-rank	1	(1)
Other	6	(7)

^a^Time-to-event outcome reported as Continuous Data

^b^For trials reporting a statistical method and a non-inferiority/equivalence hypothesis, the method was recorded

^c^Trial NCT01132118 reported two statistical analyses: “Wilcoxon (Mann-Whitney)” and “Regression, Linear” for the first primary outcome

When analyzing the first primary outcome in each record for “Reporting Groups,” 84% (105/124) of primary outcomes were reported by intervention, similar to that in parallel group trials whereas 5% (6/124) were reported by sequence and two (2%) were reported by both sequence and period but in slightly different format: presented with sequence as columns and periods/time points as rows [25] or presented with period as columns and sequence as rows [13] (Table 1).

### “Serious Adverse Events”

Eighty percent (99/124) of trials reported adverse event information by intervention only, 6% (8/124) of trials reported adverse events by sequence, and 3% (4/124) reported by total only (Table 1). One trial registered both by intervention and period [38], and five registered adverse events by “other” approaches. Five trials (4%) did not report any adverse events.

## Discussion

The ClinicalTrials.gov results database is intended to support reporting of clinical trial data in a manner that is consistent with the sponsor’s pre-specified protocol and analysis plan [9, 39]. In this study, we found variation in how crossover design trials were registered on ClinicalTrials.gov. We found that many records labeled as “Crossover Assignment” in the intervention model did not use a randomized crossover design to assign interventions experimentally; instead, these trials allowed interventions to change during the course of the trial. This suggests that it could be useful to clarify whether this option in ClinicalTrials.gov should be limited to cases in which participants are assigned to a specific crossover design as an experiment as compared to designs in which participants switch to a different intervention (or non-randomized “crossing over”).

In the majority of crossover trials analyzed, the design was clearly described in the protocol section (Arms and Assigned Interventions) and the Participant Flow module of the results section. However, the reporting of results in Baseline Characteristics, Outcome Measures, and Adverse Events generally did not appear to fully reflect the crossover design, which limits the ability of others to understand important aspects of the trial results provided on ClinicalTrials.gov. As such, we provide further considerations and discussion on potential best practices for reporting results on ClinicalTrials.gov so that the trial conduct and analysis plan fully exploit the advantages of the randomized crossover trial design. These recommendations are likely to be applicable to crossover trials of other phases and other designs such as incomplete block designs. Furthermore, given the relationship between reporting results on ClinicalTrials.gov and reporting results in journal publications, this research was used to support the development of the CONSORT extension to crossover trials [40, 41] and these recommendations may be considered complementary to those expressed in the extension.

### Summary protocol information—describe the treatment sequences and intervention(s) to which the study participants are assigned in each period

**Fig. 3:**
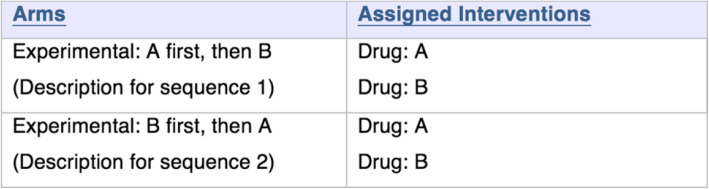
A proposal for reporting “Arms” and “Assigned Interventions” for randomized crossover trials registered on ClinicalTrials.gov

In a typical AB|BA crossover design trial, each arm is the specific sequence of interventions (“A first, then B” and “B first, then A”) to which a group of participants is randomly assigned, as depicted in the schema in Fig. 2 [6]. Best practice for describing an AB|BA crossover design trial in ClinicalTrials.gov is presented in Fig. 3, in which each arm refers to one possible sequence of interventions.

### Report Participant Flow information by sequence and by period

**Fig. 4:**
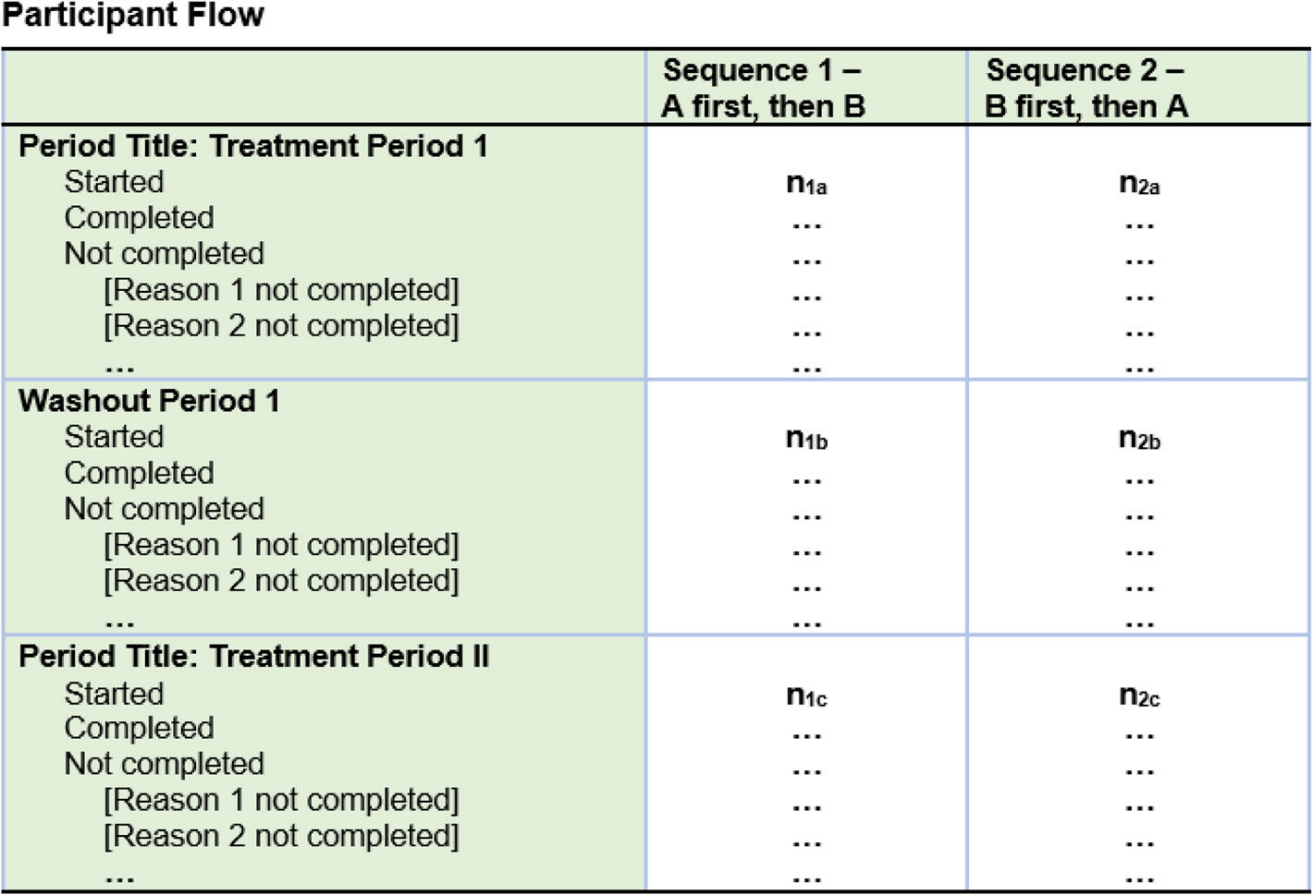
A proposal for formatting “Participant Flow” for randomized crossover trials on ClinicalTrials.gov

Similar to the protocol section, each arm in the Participant Flow module is used to represent the assignment groups in a trial. In the case of a randomized crossover design, each arm is the specific sequence of interventions to which participants were assigned and allows for understanding the number of participants starting and completing a trial. Because missing data is a threat to the design, it is particularly important to describe the number of participants at the beginning of each treatment period, with separate tables for each period to capture participant flow throughout different periods. In cases where there are no dropouts in or between periods, a single table for the entire study can provide sufficient participant flow information. Other pre-randomization periods such as open-label, run-in, and run-out phases can be described as part of pre-assignment information and washout periods can also be described as part of the participant flow. An approach for reporting Participant Flow is shown in Fig. 4.

### Report Baseline Characteristics by sequence and by period

**Fig. 5:**
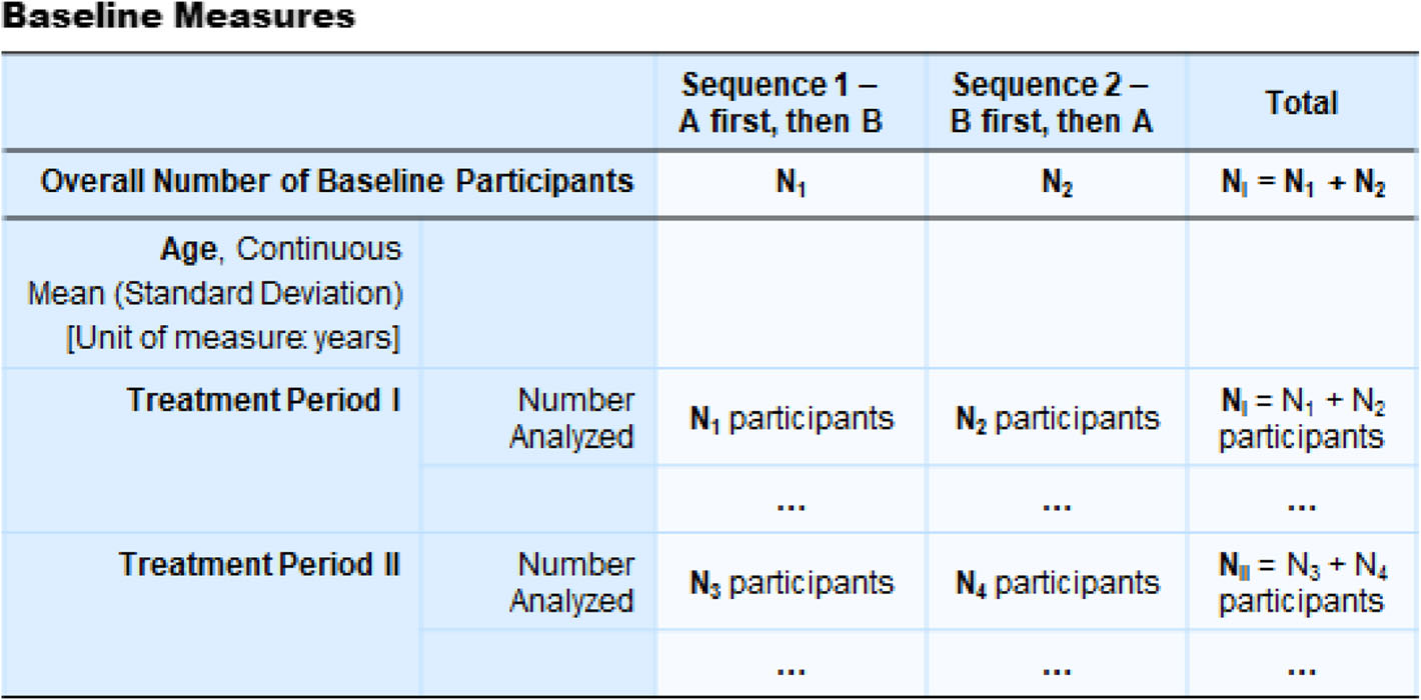
A proposal for formatting arms to report “Baseline Characteristics” for randomized crossover trials registered on ClinicalTrials.gov

In a randomized crossover trial, presenting baseline characteristics by sequence allows the assessment of the distribution of important prognostic factors between randomized sequences. It would also be useful to present this table by study period to examine whether modifiable, treatment effect-related characteristics have returned to their initial state at the beginning of the next period, which is useful for the assessment of potential period effect. An approach for reporting Baseline Characteristics is shown in Fig. 5.

### Report explicitly the analytical methods in the “Statistical analysis” section so that the users of information can understand whether the analysis accommodates the paired nature of the design

If a trial is identified as using a randomized crossover design, additional data are needed about the analysis method, specifically how the analysis accounted for repeated measures and whether the model included terms for period effects or a treatment by period interaction.

### Report Outcome Measures in a way that accounts for the design, as well as other relevant data to facilitate understanding of any carryover effect, period effect, and missing data

**Fig. 6:**
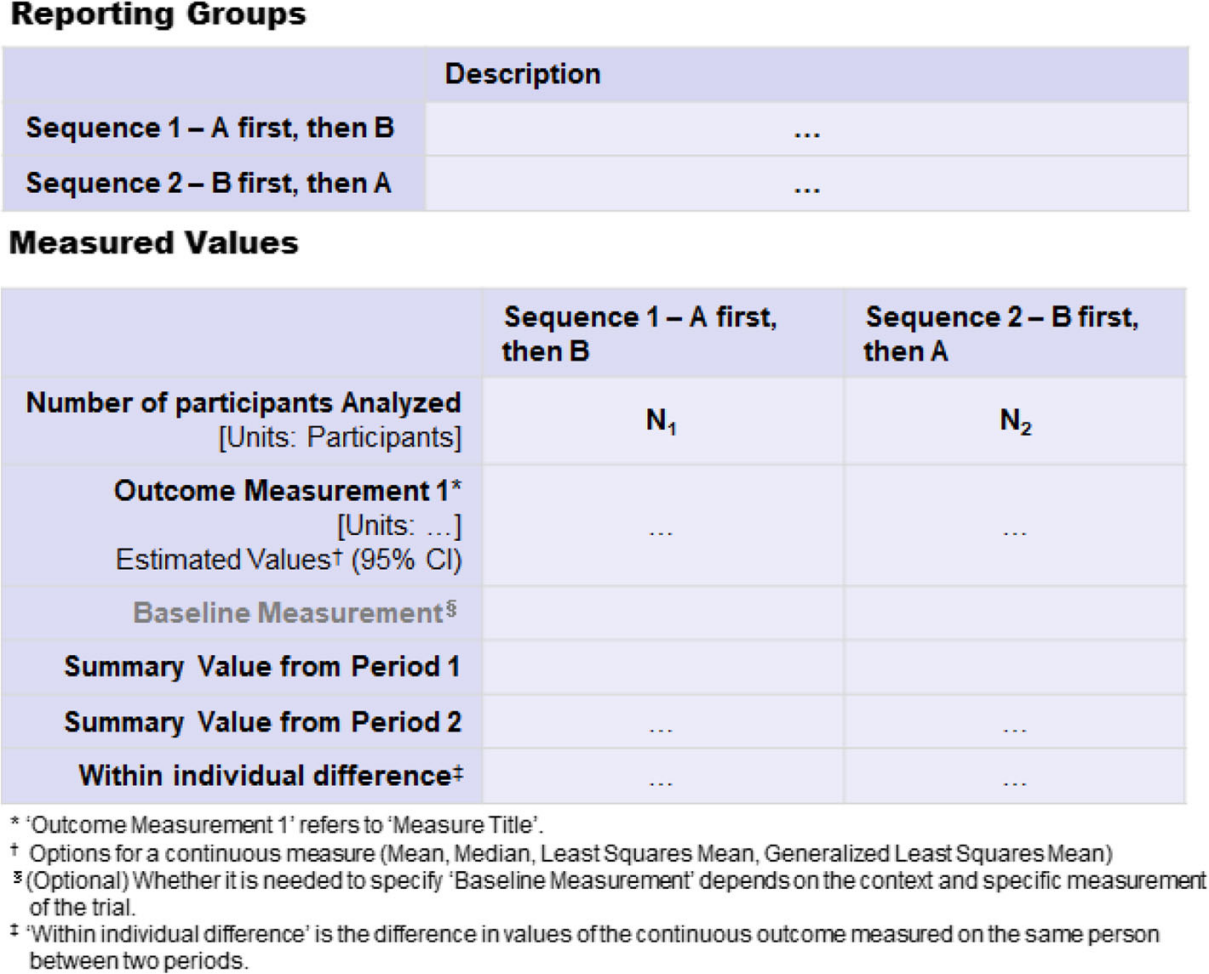
A proposal for reporting a continuous outcome for randomized crossover trials registered on ClinicalTrials.gov

The analysis of outcome data in a randomized crossover design trial should account for the correlation of repeated measurements in the same individual. In addition, period level data are useful to understand whether there is any carryover or period effect as well as the extent of missing data for a specific outcome [6]. Building upon a previous study with an example of reporting a continuous outcome for an AB|BA crossover trial [6], a similar approach is described in Fig. 6 for reporting outcome measures on ClinicalTrials.gov.

**Fig. 7:**
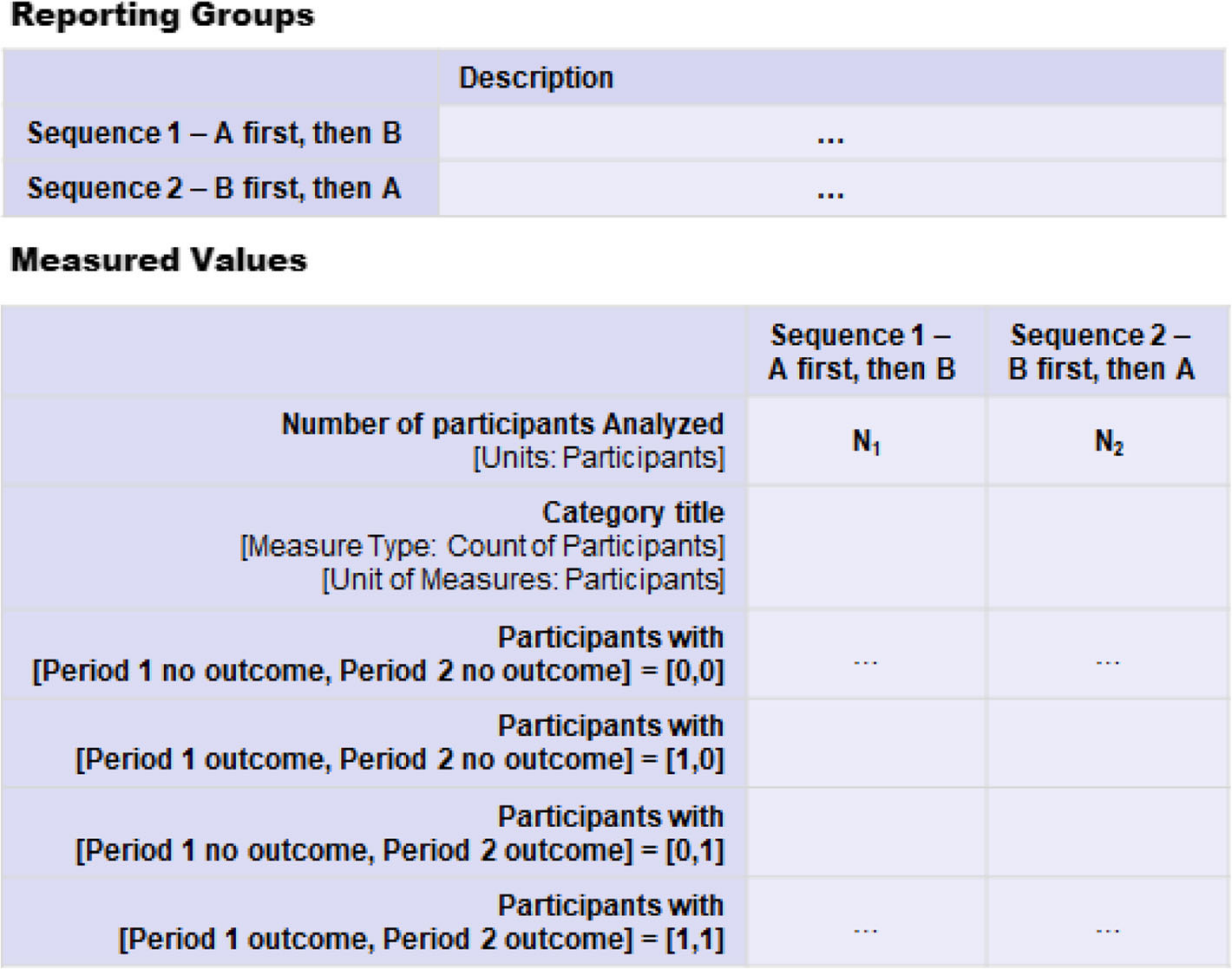
A proposal for reporting a binary outcome for randomized crossover trials registered on ClinicalTrials.gov

Similar approaches could be applied to categorical data. Figure 7 illustrates a reporting format for binary outcome for an AB|BA crossover-design trial, where event counts from each period and each sequence, as well as the concordant and discordant counts, are listed in the measured values table accordingly. This allows the user to estimate treatment effects that account for the design [5]. Of note, when statistical models are applied to adjust for possible period and carryover effects (or other covariates), the raw counts presented in this way may be less useful.

We found five trials that registered a time-to-event outcome among our sample [23, 30, 42–44], an example is shown in S Figure 3 [23]. Further evaluation is needed to provide a recommendation for optimizing the reporting of time-to-event data on ClinicalTrials.gov.

### Report Adverse Events by intervention and period

To describe Adverse Events and properly account for the design and the repeated measurements, one way of reporting is to present the analysis of adverse events by intervention and by periods, an example is shown in S Figure 4 [38]. However, this way of reporting does not allow estimation of risk of adverse events attributable to an intervention.

### Limitations

There are several limitations with this study. We chose phase 3 trials as our first step to characterizing issues in reporting of crossover design trials on ClinicalTrials.gov. We expect that phase 1 and 2 trials have similar problems. In evaluating Outcome Measures, we focused on the first listed primary outcome when more than one primary outcome was reported. Additionally, we restricted our sample to registries with a linked publication to allow for verification of unclear items in the registration, but we may have excluded some trials which had associated publications that were not linked.

## Conclusions

In conclusion, the registration and reporting of randomized crossover trials must account for the paired nature of the design. Our observations and recommendations informed the development of guidelines for good reporting practices in the registration and reporting of randomized crossover trials [40, 41].

## Supplementary information

**Additional file 1:S Table 1**. Characteristics of phase 3 randomized crossover trials registered on ClinicalTrials.gov (N = 124). **S Figure 1.** Examples of registering ‘Arms’ and ‘Assigned Interventions’. **S Figure 2.** Examples of registering ‘Participant Flow’. **S Figure 3**. An example of registering a time-to-event outcome (NCT00004635). **S Figure 4**. An example of reporting adverse events by intervention and by period (NCT00518531). **S Table 2**. Phase 3 randomized crossover trials registered on ClinicalTrials.gov (N = 124).

## Data Availability

The dataset used during the current study are available from the corresponding author on reasonable request.

## Abbreviations

SRDR: Systematic Review Data Repository
