# Supplementary material for: Registration of phase 3 crossover trials on ClinicalTrials.gov
Source: Trials. 2020 Jul 6;21:613. doi: 10.1186/s13063-020-04545-2 (PMC7336618; doi:10.1186/s13063-020-04545-2)
Supplement: Supplementary file 1 — Additional file 1:S Table 1. Characteristics of phase 3 randomized crossover trials registered on ClinicalTrials.gov (N = 124). S Figure 1. Examples of registering ‘Arms’ and ‘Assigned Interventions’. S Figure 2. Examples of registering ‘Participant Flow’. S Figure 3. An example of registering a time-to-event outcome (NCT00004635). S Figure 4. An example of reporting adverse events by intervention and by period (NCT00518531). S Table 2. Phase 3 randomized crossover trials registered on ClinicalTrials.gov (N = 124). [file 13063_2020_4545_MOESM1_ESM.docx]

**Supplement Information**

**S Table 1 Characteristics of phase 3 randomized crossover trials registered on ClinicalTrials.gov (N = 124)**

**S Figure 1 Examples of registering ‘Arms’ and ‘Assigned Interventions’**

**S Figure 2 Examples of registering ‘Participant Flow’**

**S Figure 3 An example of registering a time-to-event outcome (NCT00004635)**

**S Figure 4 An example of reporting adverse events by intervention and by period (NCT00518531)**

**S Table 2 Phase 3 randomized crossover trials registered on ClinicalTrials.gov (N = 124)**

**S Table 1 Characteristics of phase 3 randomized crossover trials registered on ClinicalTrials.gov (N = 124)**

| **Characteristics^§^** | **n** | **(%)** |
| --- | --- | --- |
| **Study classification** |  |  |
| Efficacy Study | 60 | (48) |
| Safety/Efficacy Study | 53 | (43) |
| Safety | 2 | (2) |
| Pharmacodynamics | 6 | (5) |
| (Blank) | 3 | (2) |
| **Funding source** |  |  |
| Industry | 99 | (80) |
| Others | 25 | (20) |
| **Intervention type** |  |  |
| Drug | 110 | (89) |
| Biological | 7 | (6) |
| Device | 7 | (6) |
| **Masking** |  |  |
| Open label | 21 | (17) |
| Single blind | 5 | (4) |
| Double blind* | 74 | (60) |
| Triple blind | 9 | (7) |
| Quadruple blind | 15 | (12) |
| ^§^ The categories for each trial characteristic are provided in the registration system for ‘Protocol’ section on ClinicalTrials.gov.  *Double blind refers to a type of masking where two or more parties are blinded from assignment in a trial, based on glossary on ClinicalTrials.gov. | | |

**S Figure 1 Examples of registering ‘Arms’ and ‘Assigned Interventions’**

1. An example of registering ‘Arms’ and ‘Assigned Interventions’ by sequence (NCT00090142)


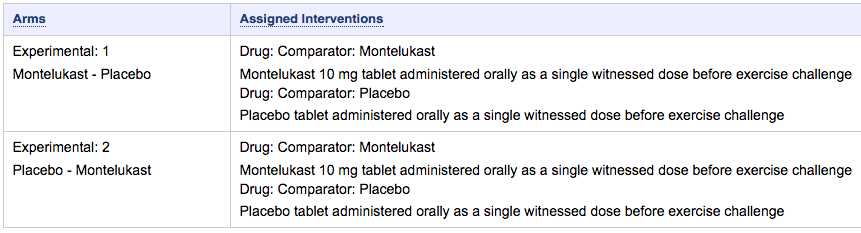


Sequence2

Sequence1

1. An example of registering ‘Arms’ by intervention (NCT00690820)


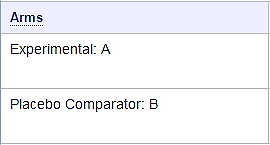


Intervention 1

Intervention 2

**S Figure 2 Examples of registering ‘Participant Flow’**

1. An example of registering ‘Participant Flow’ using one table (NCT00432744)

*
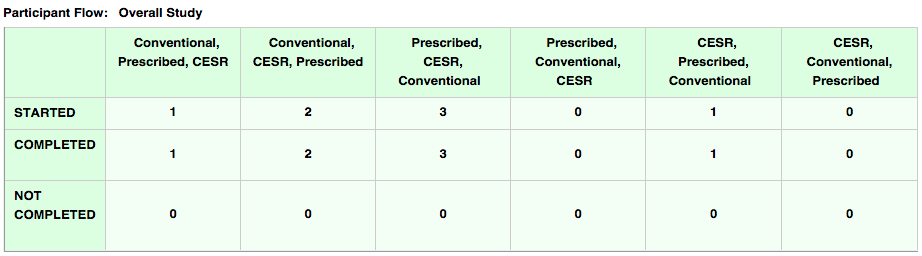
*

1. An example of registering ‘Participant Flow’ using separate tables for different periods (NCT00432744) [28]

*
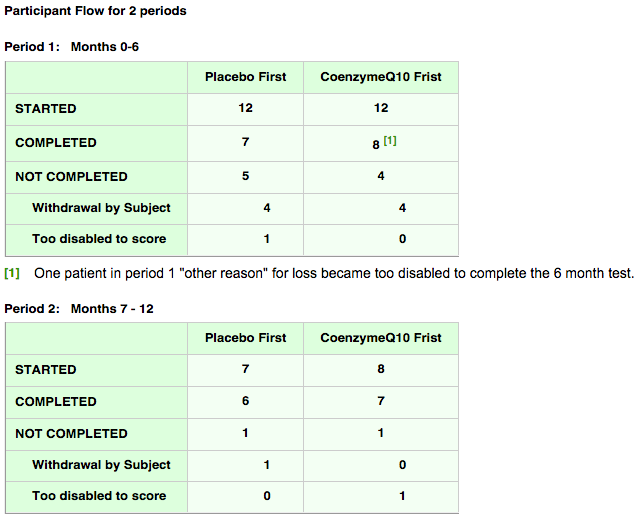
*

**S Figure 3 An example of registering a time-to-event outcome (NCT00004635)**


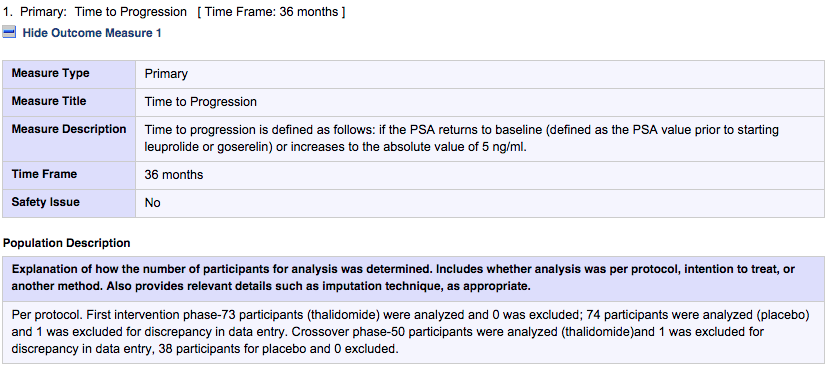


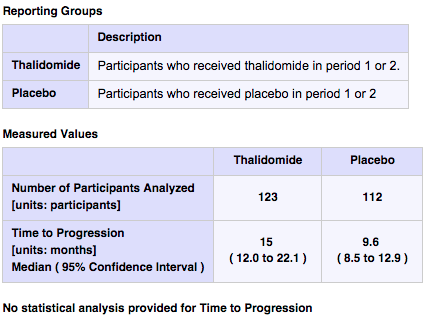

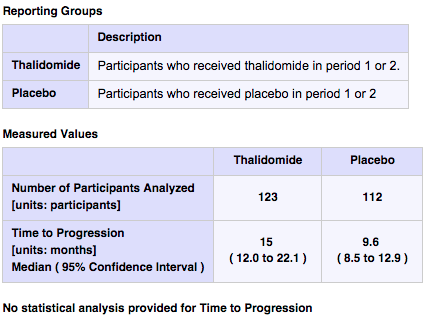

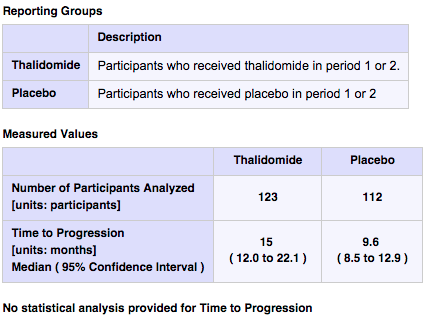


**S Figure 4 An example of reporting adverse events by intervention and by period (NCT00518531)**

**
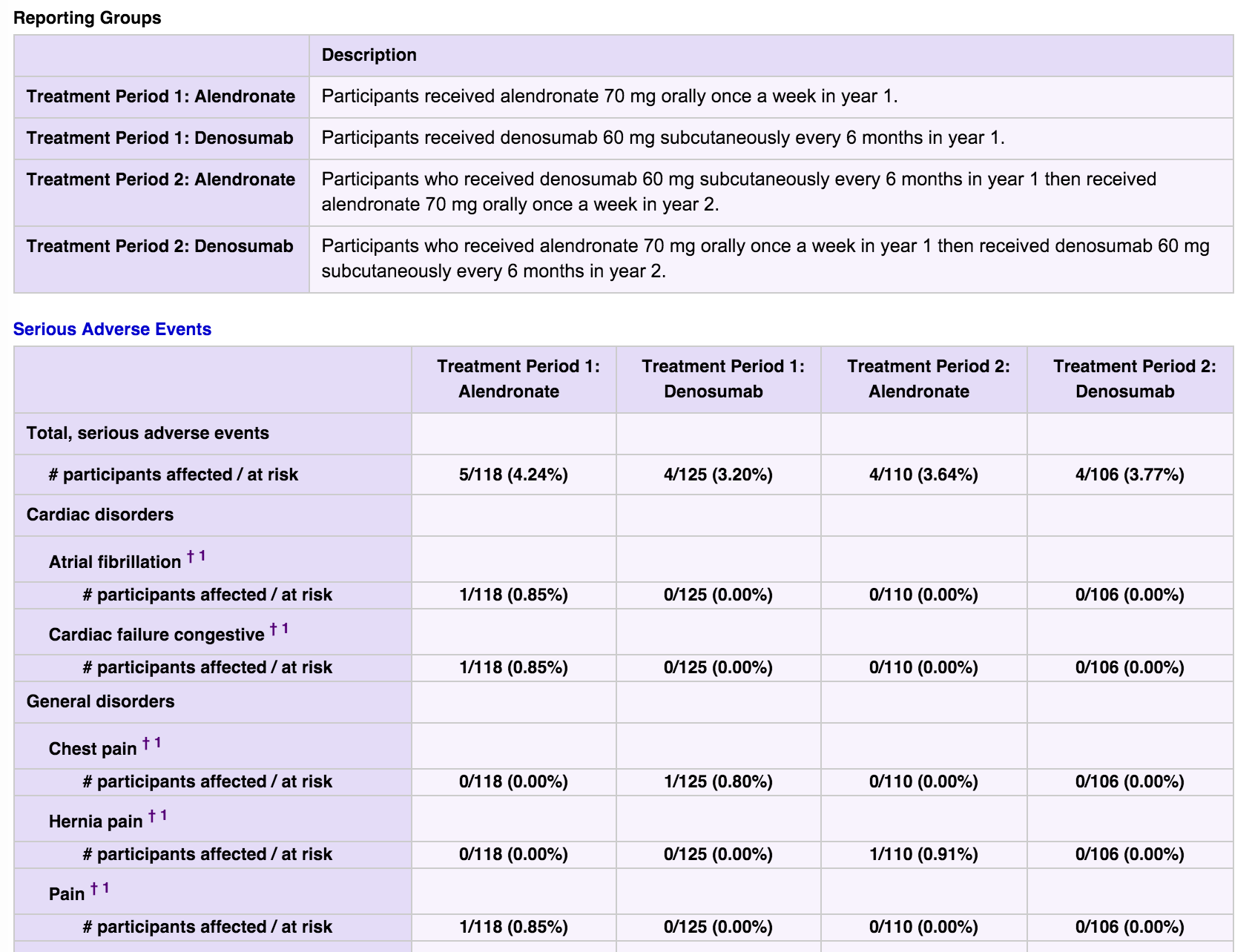
**

* Only first few adverse events the trial reported on ClinicalTrials.gov are listed as an example above.

**S Table 2 Phase 3 randomized crossover trials registered on ClinicalTrials.gov (N = 124)**

| **ID** | **ClinicalTrials.gov #** | **Title** |
| --- | --- | --- |
| 1 | NCT00004635 | Thalidomide for the Treatment of Hormone-Dependent Prostate Cancer |
| 2 | NCT00090142 | Montelukast in Exercise-Induced Bronchospasm - 2004 (0476-275)(COMPLETED) |
| 3 | NCT00092131 | Montelukast in Exercise-Induced Bronchospasm - 2003 (0476-270) |
| 4 | NCT00127166 | Two Investigational Drugs in the Prevention of Airway Constriction Brought on by Exercise in Asthmatic Patients (0476-911) |
| 5 | NCT00131248 | Medical Treatment for Gastroesophageal Reflux Disease (GERD) in Preterm Infants |
| 6 | NCT00200967 | Asthma Clinical Research Network (ACRN) Trial - Long-Acting Beta Agonist Response by Genotype (LARGE) |
| 7 | NCT00245570 | Montelukast Compared With Placebo and Salmeterol in Exercise-Induced Bronchoconstriction (0476-316) |
| 8 | NCT00276016 | The Effects of Phenylephrine Compared With Those of Placebo and Pseudoephedrine on Nasal Congestion in Subjects With Seasonal Allergic Rhinitis (SAR)(P04579) |
| 9 | NCT00295061 | Comparison of Pharmacokinetic, Safety, Tolerability of Alpha-1 MP and Prolastin In Alpha1-antitrypsin Deficient Adults (ChAMP) |
| 10 | NCT00297167 | Study to Evaluate the Safety and Efficacy of EUR-1008 (APT-1008) Pancreatic Enzyme Product in Participants With Cystic Fibrosis and Exocrine Pancreatic Insufficiency |
| 11 | NCT00322231 | A Study of an Investigational Zoster Vaccine, in Subjects With a History of Herpes Zoster (V211-014) |
| 12 | NCT00364182 | Study Comparing On-Demand Treatment With Two Prophylaxis Regimens Of BeneFIX In Patients With Severe Hemophilia B |
| 13 | NCT00382993 | A Study of Combination Product (Sumatriptan Succinate and Naproxen Sodium) in Migraine Subjects Who Report Poor Response or Intolerance to Short Acting Triptans (Study 2 of 2) |
| 14 | NCT00383162 | A Study of Combination Product (Sumatriptan Succinate and Naproxen Sodium) in Migraine Subjects Who Report Poor Response or Intolerance to Short Acting Triptans (Study 1 of 2) |
| 15 | NCT00393042 | Sleep and Tolerability Study: Comparing the Effects of Adderall XR and Focalin XR |
| 16 | NCT00395304 | Childhood Asthma Research and Education (CARE) Network Trial - Best Add-On Therapy Giving Effective Response (BADGER) |
| 17 | NCT00432744 | Phase III Trial of Coenzyme Q10 in Mitochondrial Disease |
| 18 | NCT00432835 | Temporary Gastric Electrical Stimulation for Drug Refractory Gastroparesis |
| 19 | NCT00441545 | Head to Head Study Against Sevelamer Hydrochloride |
| 20 | NCT00487695 | Confocal Endomicroscopy for Barrett's Esophagus |
| 21 | NCT00489411 | Duloxetine in Treating Peripheral Neuropathy Caused by Chemotherapy in Patients With Cancer |
| 22 | NCT00494143 | Metabolic Cost Savings for Transtibial Amputees Wearing the Controlled Energy Storage and Return (CESR) Foot |
| 23 | NCT00500149 | A Classroom Study to Assess the Time of Onset of Vyvanse (Lisdexamfetamine Dimesylate) in Pediatric Subjects Aged 6-12 With Attention Deficit/Hyperactivity Disorder (ADHD) |
| 24 | NCT00506285 | Methylphenidate Transdermal System (MTS) in the Treatment of Adult ADHD |
| 25 | NCT00518531 | Denosumab Adherence Preference Satisfaction Study |
| 26 | NCT00522951 | SH L 562BB Phase II/III Dose Justification and Gadoteridol-controlled Comparative Study |
| 27 | NCT00538850 | Fentanyl Sublingual Spray in Treating Patients With Breakthrough Cancer Pain |
| 28 | NCT00554853 | PPAR-gamma Agonists, Rheumatoid Arthritis and Cardiovascular Disease (RAPPAR) |
| 29 | NCT00565266 | Asthma Clinical Research Network (ACRN) Trial - Tiotropium Bromide as an Alternative to Increased Inhaled Corticosteroid in Patients Inadequately Controlled on a Lower Dose of Inhaled Corticosteroid (TALC) |
| 30 | NCT00572572 | Aprepitant + a 5HT3 + Dexamethasone in Patients With Germ Cell Tumors |
| 31 | NCT00573170 | TREXIMET Versus Butalbital-containing Combination Medications for the Acute Treatment of Migraine in Adults |
| 32 | NCT00574548 | Study Evaluating the Safety, Tolerability and Immunogenicity of 13vPnC as a 2-Dose Regimen or With 23vPS |
| 33 | NCT00594516 | Study on the Safety and Effectiveness of Switching Between Two Forms of Tapentadol in Patients With Chronic Low Back Pain |
| 34 | NCT00600028 | Treatment of Chronic Cough in Idiopathic Pulmonary Fibrosis With Thalidomide |
| 35 | NCT00615030 | Study of Indacaterol Dosed in the Evening in Patients With Chronic Obstructive Pulmonary Disease (COPD) |
| 36 | NCT00615459 | A Crossover Study to Determine the Effect on Lung Function of Indacaterol in Patients With Moderate to Severe Chronic Obstructive Pulmonary Disease (COPD), Using Tiotropium as an Active Control |
| 37 | NCT00620022 | The Effect of Indacaterol on Exercise Endurance in Patients With Moderate to Severe Chronic Obstructive Pulmonary Disease |
| 38 | NCT00626028 | Comparison of Inhaled Nitric Oxide and Oxygen in Patient Reactivity During Acute Pulmonary Vasodilator Testing |
| 39 | NCT00660075 | Effects of Sitagliptin on Postprandial Lipemia in Men With Type 2 Diabetes |
| 40 | NCT00662818 | Telcagepant (MK-0974) Treatment of Migraine in Participants With Stable Vascular Disease (MK-0974-034) |
| 41 | NCT00666263 | Study of the Effectiveness of Intravenous Immune Globulin (10%) for the Treatment of Multifocal Motor Neuropathy |
| 42 | NCT00667992 | Study Comparing Budesonide Hydrofluoroalkane (HFA) vs Chlorofluorocarbon (CFC) Pressurized Metered Dose Inhalers (pMDI) in Patients With Mild to Moderate Asthma |
| 43 | NCT00690820 | Study Investigating a Delayed-Release Pancrelipase in Patients With Pancreatic Exocrine Insufficiency (PEI) Due to Cystic Fibrosis (CF) |
| 44 | NCT00697515 | Safety and Efficacy Workplace Environment Study of Lisdexamfetamine Dimesylate (LDX) in Adults With Attention-Deficit Hyperactivity Disorder (ADHD) |
| 45 | NCT00734604 | A Study for Patients With Erectile Dysfunction to Test Whether Tadalafil Taken Once a Day Can Better Improve Psychological Outcomes. |
| 46 | NCT00748098 | Polysomnography Study of GSK1838262 Extended Release Tablets Versus Placebo in RLS and Associated Sleep Disturbance |
| 47 | NCT00773279 | Efficacy, Safety and Preference Study of a Insulin Pen PDS290 vs. a Novo Nordisk Marketed Insulin Pen in Diabetics |
| 48 | NCT00812006 | A Study of Rizatriptan for the Treatment of Acute Migraine in Patients on Topiramate for Migraine Prophylaxis |
| 49 | NCT00813488 | Fentanyl Buccal Tablets Versus Immediate Release Oxycodone for Breakthrough Pain in Patients With Chronic Pain |
| 50 | NCT00837967 | Study to Investigate the Safety and Efficacy of High Dose of Symbicort SMART in Japanese Patients |
| 51 | NCT00883740 | Effects Of Pregabalin (Lyrica) On Sleep Maintenance In Subjects With Fibromyalgia |
| 52 | NCT00894556 | A Study to Evaluate the Efficacy and Tolerability of Rizatriptan for Treatment of Acute Migraine (0462-087) |
| 53 | NCT00904670 | Quillivant Oral Suspension (Quillivant XR) in the Treatment of Attention Deficit Hyperactivity Disorder (ADHD) |
| 54 | NCT00931385 | Characterization of 24 Hour Spirometry Profiles of Inhaled BI 1744 CL and Inhaled Foradil in Patients With Chronic Obstructive Pulmonary Disease |
| 55 | NCT00932646 | Characterisation of 24-hour FEV1-time Profiles of Inhaled BI 1744 CL and Inhaled Foradil in Patients With Chronic Obstructive Pulmonary Disease |
| 56 | NCT00991276 | Polysomnography Study Of Pregabalin And Pramipexole Versus Placebo In Patients With Restless Legs Syndrome And Associated Sleep Disturbance |
| 57 | NCT00992459 | Study of the Efficacy and Safety of HPN-100, Glyceryl Tri-(4-phenylbutyrate), for the Treatment of Adults With Urea Cycle Disorders (Help UCD) |
| 58 | NCT00999908 | Comparison of the Effects of Indacaterol and Tiotropium on Inspiratory Capacity |
| 59 | NCT01000961 | Phase 3 Study of Cysteamine Bitartrate Delayed-release (RP103) Compared to Cystagon in Patients With Cystinosis |
| 60 | NCT01005888 | C1 Esterase Inhibitor (C1INH-nf) for the Prevention of Acute Hereditary Angioedema (HAE) Attacks |
| 61 | NCT01012765 | Effect of Indacaterol on Inspiratory Capacity (IC) |
| 62 | NCT01029340 | Trial to Evaluate the Efficacy and Safety of a New Full Length Recombinant Human FVIII for Hemophilia A (Leopold I) |
| 63 | NCT01040130 | Effect of Treatment BI 1744 CL (5 and 10 mcg) Versus Placebo on Exercise Endurance Time During Constant Work Rate Cycle Ergometry I |
| 64 | NCT01040793 | Effect of Treatment BI 1744 CL (5 and 10 mcg) Versus Placebo on Exercise Endurance Time During Constant Work Rate Cycle Ergometry II |
| 65 | NCT01064310 | Patient Preference Study of Pazopanib Versus Sunitinib in Advanced or Metastatic Kidney Cancer |
| 66 | NCT01072149 | A Study to Evaluate the Efficacy, Safety, Tolerability, Pharmacokinetic (PK), and Pharmacodynamic (PD) Profiles of 3 Doses of Fluticasone Furoate (FF)/GW642444 Inhalation Powder at the End of a 28-day Treatment Period in Subjects With Chronic Obstructive Pulmonary Disease (COPD) Compared to Placebo |
| 67 | NCT01112865 | Cross Over Convenience And Preference Study Of New Mark VII Compared To Genotropin Pen In Pediatric And Adult Subjects (CHOOSE) |
| 68 | NCT01132118 | Hydroxychloroquine to Improve Insulin Sensitivity in Rheumatoid Arthritis |
| 69 | NCT01154127 | Effect of NVA237 on Exercise Endurance in Patients With Chronic Obstructive Pulmonary Disease (COPD) |
| 70 | NCT01233258 | A Trial to Compare Prophylaxis Therapy to On-demand Therapy With a New Full Length Recombinant FVIII in Patients With Severe Hemophilia A (Leopold II) |
| 71 | NCT01273883 | A Trial of Magnesium Dependent Tinnitus |
| 72 | NCT01294787 | Effect of QVA149 on Exercise Tolerance in Patients With Chronic Obstructive Pulmonary Disease (COPD) |
| 73 | NCT01370590 | A Study to Evaluate the Effectiveness of Ezetimibe/Atorvastatin 10 mg/20 mg Combination Tablet Compared to Marketed Ezetimibe 10 mg and Atorvastatin 20 mg Tablets in Participants With High Cholesterol (MK-0653C-185 AM1) |
| 74 | NCT01370603 | A Study to Evaluate the Effectiveness of Ezetimibe/Atorvastatin 10 mg/40 mg Combination Tablet Compared to Marketed Ezetimibe 10 mg and Atorvastatin 40 mg Tablets in Participants With High Cholesterol (MK-0653C-190 AM1) |
| 75 | NCT01401465 | Study To Evaluate Patient Preference, Satisfaction And Efficacy Of a Nasal Aerosol Versus an Aqueous Nasal Spray |
| 76 | NCT01432236 | A Phase 3b Multicenter Study of Pregabalin in Fibromyalgia Subjects Who Have Comorbid Depression |
| 77 | NCT01455415 | Effect Of Pregabalin Treatment In Patients With Diabetic Nerve Pain Who Currently Use A Non-Steroid Anti-Inflammatory Drug (NSAID) For Another Pain |
| 78 | NCT01462370 | Study to Assess the Safety and Efficacy of Etoricoxib Versus Ibuprofen in the Treatment of Dysmenorrhea (MK-0663-145 AM1) |
| 79 | NCT01471171 | Efficacy and Safety of Aclidinium Bromide 400 g BID (Twice a Day)Compared to Placebo in Patients With Stable Moderate to Severe Chronic Obstructive Pulmonary Disease (COPD) |
| 80 | NCT01474772 | Efficacy and Safety Study of Pregabalin in the Treatment of Pain on Walking in Patients With Diabetic Peripheral Neuropathy (DPN) |
| 81 | NCT01490125 | The Effect of QVA149 on Patient Reported Dyspnea in Moderate to Severe Chronic Obstructive Pulmonary Disease (COPD) |
| 82 | NCT01515657 | Pharmacodynamic Evaluation of PL2200 Versus Enteric-Coated and Immediate Release Aspirin in Diabetic Patients |
| 83 | NCT01533922 | Effect on Exercise Endurance and Lung Hyperinflation of Tiotropium + Olodaterol in COPD Patients |
| 84 | NCT01533935 | Effect on Exercise Endurance and Lung Hyperinflation of Tiotropium + Olodaterol in COPD Patients. |
| 85 | NCT01559012 | Transdermal Clonidine in the Treatment of Severe Hyperemesis Gravidarum (CLONEMESI) |
| 86 | NCT01570751 | A Trial Comparing the Efficacy, Patient-reported Outcomes and Safety of Insulin Degludec 200 U/mL vs Insulin Glargine in Subjects With Type 2 Diabetes Mellitus Requiring High-dose Insulin |
| 87 | NCT01606189 | A Study to Compare Sublingual Cannabis Based Medicine Extracts With Placebo to Treat Brachial Plexus Injury Pain |
| 89 | NCT01606306 | Individualized Therapy For Asthma in Toddlers (INFANT) |
| 90 | NCT01607411 | A Clinical Study to Evaluate Experimental Children's Toothpastes in an In-Situ Caries Model |
| 91 | NCT01667679 | Efficacy and Safety of 20 mg Sumatriptan Powder Delivered Intranasally With the Bi-directional Device Compared With 100 mg Sumatriptan Tablets in Adults With Acute Migraine With or Without Aura (COMPASS) |
| 92 | NCT01691885 | RELOVAIR Lung Deflation Study |
| 93 | NCT01699685 | Swiss studY for the Treatment of COPD Patients With the Free combiNation of indacatERol and GlYcopyrroniumbromide. (SYNERGY) |
| 94 | NCT01754259 | Effects of Ranolazine on Coronary Flow Reserve in Symptomatic Diabetic Patients and CAD (RAND-CFR) |
| 95 | NCT01766076 | Atorvastatin for HAART Suboptimal Responders |
| 96 | NCT01791972 | Efficacy of Albuterol SPIROMAX in Adult and Adolescent Patients With Exercise-Induced Bronchoconstriction (EIB) |
| 97 | NCT01808755 | D Mannose in Recurrent Urinary Tract Infections |
| 98 | NCT01829243 | Milnacipran and Neurocognition, Pain and Fatigue in Fibromyalgia : A 13-week Randomized, Placebo Controlled Cross Over Trial |
| 99 | NCT01835912 | Chronic Versus Acute Dosing of Sodium Citrate for Swimming 200m |
| 100 | NCT01857063 | Study of the Efficacy and Safety of MK-0476 in Japanese Pediatric Participants With Seasonal Allergic Rhinitis (MK-0476-519) |
| 101 | NCT01868009 | DISKUS vs. ELLIPTA Device Preference Study in Chronic Obstructive Pulmonary Disease (COPD) |
| 102 | NCT01962922 | Crossover Study to Compare PK of Once Daily LCP-Tacro Tablets to Generic Tacrolimus Capsules Twice Daily. |
| 103 | NCT01967173 | Best African American Response to Asthma Drugs (BARD) |
| 104 | NCT01978119 | A Non-inferiority Study to Evaluate the Efficacy, Safety, and Tolerability of Combination Dry Powder of Fluticasone Propionate and Salmeterol (FSC) 250/50 Microgram (mcg) Twice Daily (BID) in Adults and Adolescents With Asthma |
| 105 | NCT01978145 | A Non-inferiority Study to Evaluate the Efficacy, Safety, and Tolerability of Fluticasone Propionate/Salmeterol (FSC) 250/50 Microgram (mcg) Through a Capsule-Based Inhaler and a Multi-Dose Inhaler Administered Twice Daily (BID) in Adults With Chronic Obstructive Pulmonary Disease (COPD) |
| 106 | NCT01994746 | Efficacy and Safety of Nasal Glucagon for Treatment of Hypoglycemia in Adults |
| 107 | NCT01996319 | Randomized, Double-blind, Placebo-controlled, Multicenter, Cross-over Study to Assess the Effects of a 3 Week Therapy Each With QVA149 Versus Placebo on Pulmonary Function and Average Physical Activity Levels in Patients With COPD. (MOVE) |
| 108 | NCT02030600 | A Trial Comparing the Safety and Efficacy of Insulin Degludec and Insulin Glargine, With or Without OADs in Subjects With Type 2 Diabetes (SWITCH 2) |
| 109 | NCT02034513 | A Trial Comparing the Safety and Efficacy of Insulin Degludec and Insulin Glargine, Both With Insulin Aspart as Mealtime Insulin in Subjects With Type 1 Diabetes (SWITCH 1) |
| 110 | NCT02052141 | Safety and Efficacy Study of CINRYZE for Prevention of Angioedema Attacks in Children Ages 6-11 With Hereditary Angioedema |
| 111 | NCT02066298 | Steroids In Eosinophil Negative Asthma (SIENA) |
| 112 | NCT02147158 | A Study of the Efficacy and Safety of Ulipristal Acetate Intermittent Treatment for Abnormal Uterine Bleeding Associated With Leiomyomas |
| 113 | NCT02226198 | A Study to Evaluate the Efficacy and Safety of Rosuvastatin in Children and Adolescents With Homozygous Familial Hypercholesterolemia (HYDRA) |
| 114 | NCT02275546 | Performance and Safety of Vaginal Ring Applicator in Healthy Females (MK-8342A-063) |
| 115 | NCT02322788 | Comparing the Efficacy of Bricanyl M2 and Bricanyl M3 at 0.5 and 1.5 mg Dose Levels, to Allow for a Switch From Bricanyl Turbuhaler M2 to Bricanyl Turbuhaler M3 |
| 116 | NCT02339246 | Pharmacokinetic Comparison Of All FK-506 Formulations (ASTCOFF) |
| 117 | NCT02347072 | 24-hour Lung Function in Subjects With Moderate to Very Severe COPD After Treatment With PT003, Open-Label Spiriva Respimat as an Active Control, and Placebo |
| 118 | NCT02347085 | 24-hour Lung Function in Subjects With Moderate to Very Severe COPD After Treatment With PT003 and Placebo MDI |
| 119 | NCT02487446 | Efficacy and Safety Study of QVA149 in COPD Patients |
| 120 | NCT02487498 | Efficacy and Safety Study of Indacaterol Maleate/Glycopyrronium Bromide in Chronic Obstructive Pulmonary Disease (COPD) Patients. |
| 121 | NCT02502734 | Effect of Inhaled Fluticasone Furoate on Short-term Growth in Paediatric Subjects With Asthma |
| 123 | NCT02584959 | Study to Evaluate the Clinical Efficacy and Safety of Subcutaneously Administered C1 Esterase Inhibitor for the Prevention of Angioedema Attacks in Adolescents and Adults With Hereditary Angioedema |
| 124 | NCT02629965 | Comparing the Efficacy of Tiotropium + Olodaterol Fixed Dose Combination (FDC) Over Tiotropium in Improvement of Lung Hyperinflation, Exercise Capacity and Physical Activity in Japanese COPD Patients |
